# Robotic Resection in Succinate Dehydrogenase Subunit B (SDHB)-Mutated Hereditary Paraganglioma: A Case Report of Two Patients and A Literature Review

**DOI:** 10.7759/cureus.56336

**Published:** 2024-03-17

**Authors:** Ekaterina Baron, Chih Ching Wu, Kanchan Gupta, Jessica A Wernberg, Michael T Sheehan, Rohit Sharma

**Affiliations:** 1 Surgical Oncology, Marshfield Medical Center, Wisconsin, USA; 2 Endocrinology, Marshfield Medical Center, Wisconsin, USA

**Keywords:** robotic-assisted surgery, familial paraganglioma, surveillance, postoperative follow-up, gene mutation, hereditary paraganglioma-pheochromocytoma syndrome, cascade genetic testing, minimally invasive surgery, sdhb mutation, extra-adrenal paraganglioma

## Abstract

Autosomal dominant hereditary paraganglioma-pheochromocytoma syndrome (HPPS) is a rare genetic disorder characterized by neuroendocrine tumor development associated with pathogenic variants in succinate dehydrogenase (SDH) enzyme complex genes. Particularly, HPPS linked to SDHB mutation poses a significant clinical challenge due to its association with aggressive tumor features and a high risk of malignancy. Our report underscores the diversity in the presentation of patients with SDHB-mutated paraganglioma and the feasibility of managing it with a minimally invasive surgical approach. In the first case, a 17-year-old female was diagnosed with a metabolically active, poorly differentiated extra-adrenal retroperitoneal paraganglioma that required challenging robotic resection. Cascade genetic testing revealed an SDHB mutation not only in her but also in three family members, pointing to the inherited nature of the syndrome. Conversely, the second case involves a 37-year-old male with an asymptomatic well-differentiated left paraaortic paraganglioma incidentally found during an unrelated medical examination. Robotic converted-to-open resection allowed the successful removal of the mass. Subsequent germline testing confirmed a deleterious SDHB mutation, initiating a process of familial cascade testing. Both patients remained symptom- and recurrence-free at 12 and six months, respectively. Through these cases, and supported by a literature review, we highlight the variable clinical presentations of HPPS, arising from the same genetic alteration. The successful application of minimally invasive surgical techniques, combined with genetic evaluation, emphasizes the necessity for a comprehensive, tailored approach to treatment and surveillance. This strategy not only addresses the immediate clinical needs but also fosters proactive management of at-risk family members, ensuring a multidisciplinary approach to this complex hereditary condition.

## Introduction

Paragangliomas are catecholamine-secreting neuroendocrine tumors originating from neural crest-derived cells in intra- and extra-adrenal autonomic paraganglia [1]. Sympathetic paragangliomas usually secrete catecholamines and present clinically with paroxysmal hypertension, tachycardia, sweating, and flushing [2]. Parasympathetic paragangliomas are typically asymptomatic and found incidentally or due to compressive symptoms depending upon location [2,3]. Surgical resection remains the mainstay of isolated paraganglioma treatment both controlling hormonal hypersecretion and improving survival [2]. While most paragangliomas are sporadic, over 40% are associated with heritable disorders [4,5]. Therefore, genetic counseling is recommended for all patients with newly diagnosed paragangliomas [6].

Autosomal dominant paraganglioma syndrome (PGL), also known as hereditary paraganglioma-pheochromocytoma syndrome (HPPS), while uncommon, stands out as one of the primary genetic contributors to hereditary paraganglioma [7,8]. This disorder is linked to mutations in genes encoding the succinate dehydrogenase (SDH) enzyme complex, which plays a crucial role in the mitochondrial respiratory chain and the Krebs cycle [2,5,8]. Most genes in the SDHx complex are tumor suppressors [5]. Deleterious mutations in this complex are linked to an increased risk of developing intra- and extra-adrenal paragangliomas as well as gastrointestinal stromal tumors (GIST), renal-cell carcinomas (RCC), and pituitary adenomas [2]. Thus, identifying HPPS and SDHx mutations in paraganglioma patients can significantly change postoperative surveillance for patients and prompt cascade family testing. As most paragangliomas undergo resection, these patients are commonly co-managed by surgeons, who subsequently are involved in navigating their work-up and surveillance. Therefore, greater awareness of HPPS and the need for genetic testing are paramount.

We present two cases of SDHB-mutated retroperitoneal paragangliomas in 17- and 37-year-old patients who were treated with a minimally invasive surgical approach and were subsequently confirmed by genetic testing to have HPPS.

## Case presentation

Case presentation 1

**Figure 1:**
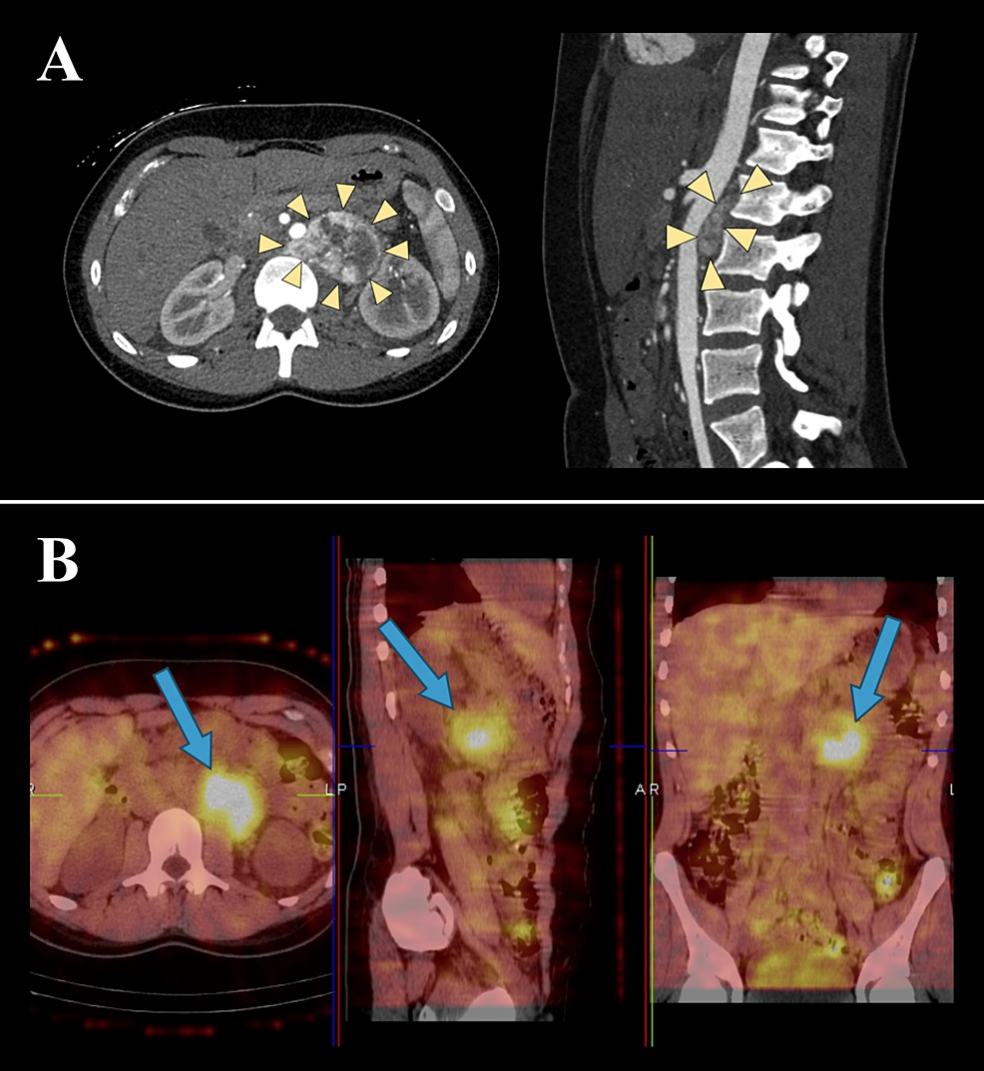
Patient 1 - contrast-enhanced CT scan of the abdomen (A) and (B) SPECT/CT scan with iodine-123 MIBG Yellow arrowheads frame the margins of the mass and blue arrows point to the diffuse activity accumulation in the mass area, separate from the left adrenal. CT, computed tomography; MIBG, meta-iodobenzylguanidine; SPECT, single-photon emission computed tomography

A 17-year-old female with a history of hypothyroidism and acne presented with severe hypertension and a one-year history of daily episodes of tachycardia, sweating, and hot flashes. Upon admission, her blood pressure was 179/96 mmHg with a heart rate of 67 beats/min. Alpha-blockade followed by beta-blockade successfully normalized the patient’s blood pressure. Plasma metanephrines and urine metanephrines and catecholamines were all significantly elevated: plasma-free normetanephrine: 12 nmol/L (normal range (NR): <0.9 nmol/L), 24-hour urine normetanephrine: 4495 ug/24hr (NR: 50-840 ug/24hr), 24-hour urine norepinephrine: 1072 ug/24hr (NR: 13-107 ug/24hr), 24-hour urine dopamine: 999 ug/24hr (NR: 20-450 ug/24hr), 24-hour urine homovanillic acid: 15.7 mg/24hr (NR: <8.0 mg/24hr), and 24-hour urine vannilymandelic acid: 21.5 mg/24hr (NR: <8.0 mg/24hr). A computed tomography (CT) scan revealed a 4.9x6.4x5.8 cm retroperitoneal tumor adjacent to the left renal hilum with a portion extending behind the abdominal aorta (Figure 1A). There were no other lesions in the chest, abdomen, or pelvis. A single-photon emission computed tomography (SPECT) scan with iodine-123 meta-iodobenzylguanidine (MIBG) demonstrated accumulation only in the mass, which was separate from the left adrenal gland, consistent with a paraganglioma (Figure 1B).

**Figure 2:**
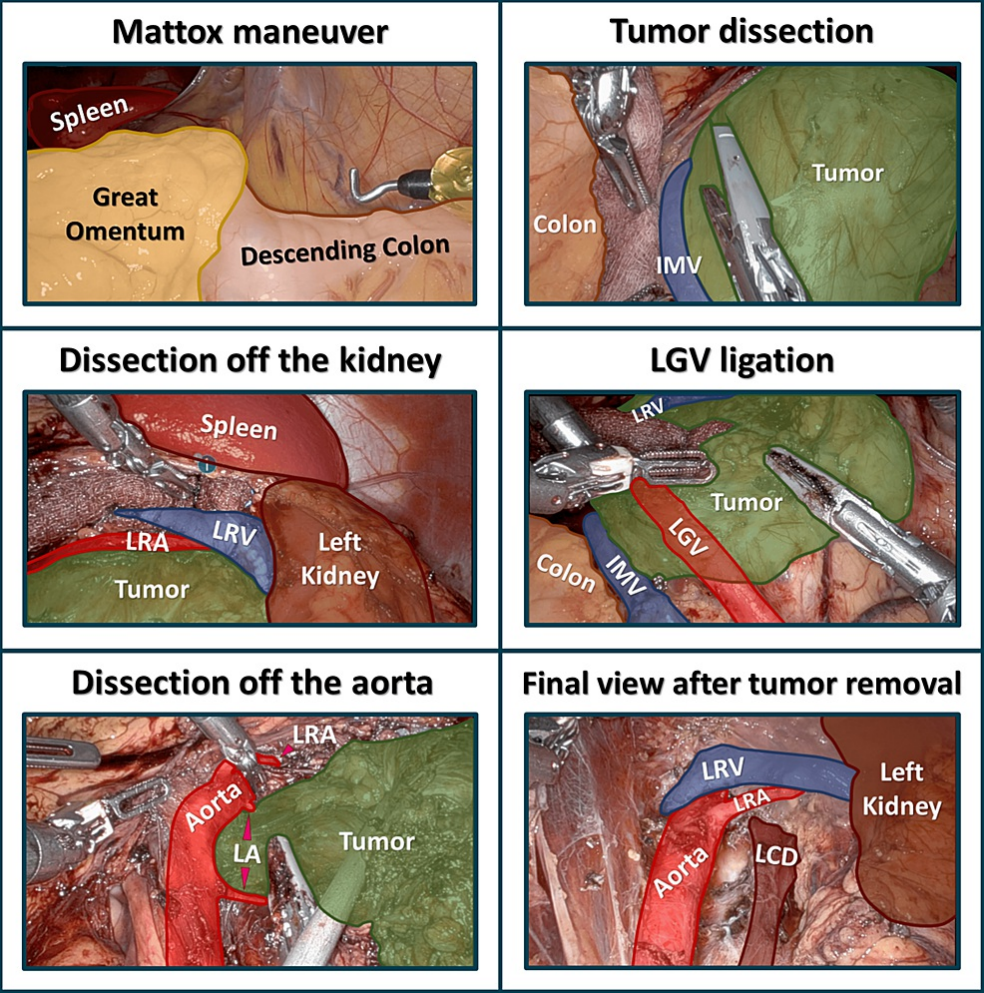
Patient 1 - key steps of robotic resection of paraganglioma with LN dissection IMV, inferior mesenteric vein; LA, lumbar arteries; LCD, left crus of the diaphragm; LGV, left gonadal vessels; LRA, left renal artery; LRV, left renal vein; LN, lymph node

**Figure 3:**
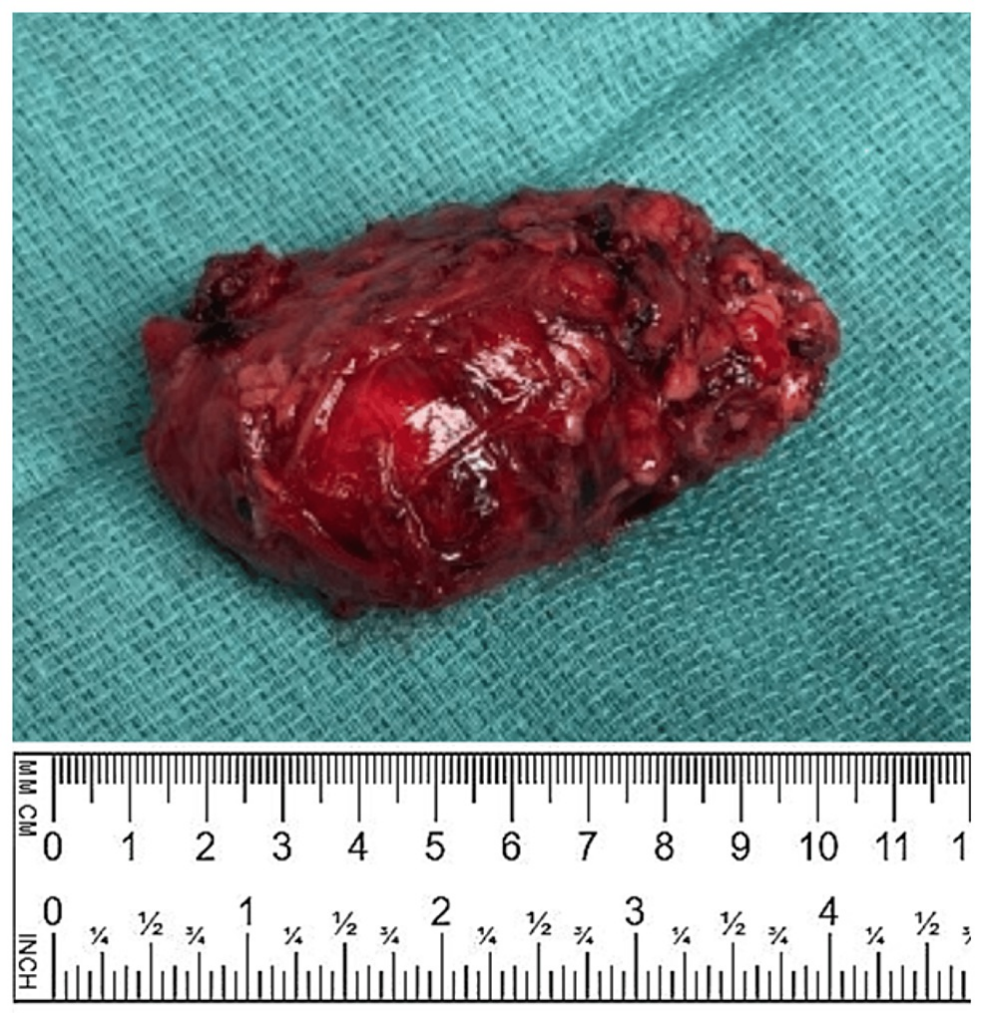
Patient 1 - surgical specimen The surgical specimen is presented as a solid, nodular, and rubbery mass, measuring 9.2x5.0x4.2 cm, encapsulated by a thin, macroscopically intact capsule. It was completely excised along with adjacent adipose tissue, including paraaortic LNs. LNs, lymph nodes

The mass and several suspicious paraaortic lymph nodes (LNs) were removed using a robotic approach (Figure 2 and Figure 3). Her postoperative course was complicated by a chylous leak, which was managed with drainage, octreotide therapy, and a low-fat diet. Pathology revealed a poorly differentiated paraganglioma with evidence of vascular invasion, a positive resection margin, and six negative LNs. Immunohistochemistry (IHC) revealed Ki67 of 7-8%, diffuse expression of chromogranin, synaptophysin, and S-100 protein, but no expression of the SDHB protein, which would typically be seen with an SDHB gene mutation. According to the grading system for adrenal pheochromocytoma and paraganglioma (GAPP), the tumor was assigned 8 out of 10 points, indicating a high-risk profile [9].

Subsequently, the patient underwent genetic testing, which identified a heterozygous pathogenic variant of SDHB c.713del confirming HPPS. Cascade genetic testing of the patient’s relatives revealed the same germline SDHB mutation in her mother, maternal uncle, and maternal grandfather. Their phenotype assessment as well as genetic testing of all their first-degree relatives is currently underway.

The patient’s circulating tumor DNA (ctDNA) test was negative one and six months after surgery. At the one-year follow-up, she remained asymptomatic with normal urine and plasma metanephrines and no CT evidence of recurrence.

Case presentation 2

A 37-year-old male presented with severe left flank pain due to nephrolithiasis and was incidentally found to have a left paraaortic mass on a CT scan. His blood pressure and heart rate were moderately elevated at 157/115 mmHg and 101 beats/min, respectively. He had no other symptoms, and his past medical history was completely unremarkable. His pain resolved after the passage of a 3 mm kidney stone.

**Figure 4:**
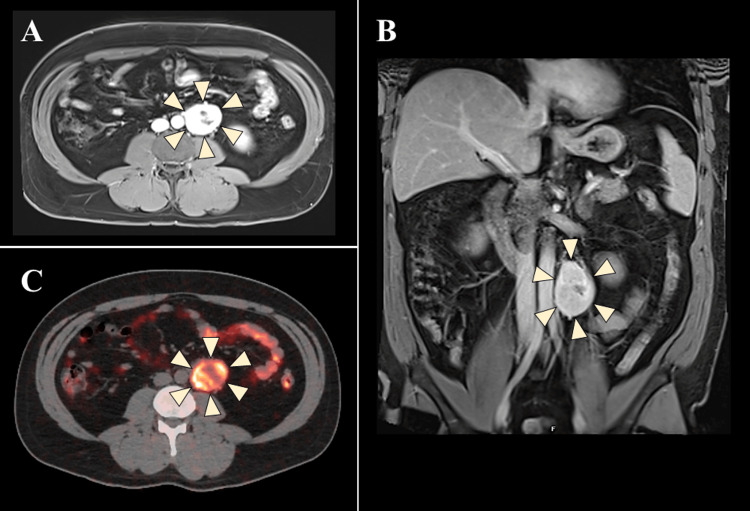
Patient 2 - MRI of the abdomen (A, B) and copper-64 Dotatate PET-CT scan (C) Yellow arrowheads frame the margins of the mass. MRI, magnetic resonance imaging; PET-CT, positron emission tomography-computed tomography

The patient was initially started on amlodipine for hypertension by his primary care provider. His plasma-free normetanephrine was mildly elevated at 1.0 nmol/L (NR: <0.9 nmol/L). His plasma-free metanephrine and 24-hour urine metanephrine and catecholamines were within NR. His antihypertensive therapy was altered to alpha-blockers with the subsequent addition of a beta-blocker. Magnetic resonance imaging confirmed a 5.2x3.4x4.1 cm enhancing, left para-aortic retroperitoneal mass (Figure 4A and Figure 4B). Copper-64 Dotatate positron emission tomography (PET)-CT demonstrated intense activity in the left paraaortic mass at the L3 level with no other lesions (Figure 4C). 

During robotic resection, the retroperitoneal mass was found to be extremely vascularized from lumbar arteries and the case was converted to an open procedure. No LNs were removed. His postoperative course was uneventful, and he was discharged on postoperative day 2. Pathology revealed a paraganglioma without vascular or capsular invasion. IHC showed diffuse expression of chromogranin, synaptophysin, and S-100 protein, and positive staining for SDHB protein. GAPP score was one out of 10, indicating low-risk features [9]. Subsequent genetic testing identified a pathogenic variant in SDHB gene c.683_684del, consistent with HPPS. Cascade family testing is currently underway. At six-month follow-up, the patient has no clinical, biochemical, or imaging evidence of recurrence. Genetic counseling has recommended further screening according to National Comprehensive Cancer Network guidelines [10].

## Discussion

In this manuscript, we report two young patients presenting with extra-adrenal paraganglioma, both of whom were found to have deleterious germline SDHB mutations resulting in familial cascade testing. These cases demonstrate the complexities and considerations needed in postoperative care and practice enhancing knowledge for surgeons operating on retroperitoneal tumors.

Paraganglioma management brings several challenges to physicians and patients. Up to 41% of paragangliomas are associated with hereditary syndromes making genetic testing obligatory for all new diagnoses as germline mutation identification can significantly alter postoperative recommendations [4-6]. Most hereditary paragangliomas are associated with PGL 1-5 caused by SDHx mutations or present as a component of syndromes such as von Hippel Lindau (VHL), multiple endocrine neoplasia (MEN) 2A and 2B, neurofibromatosis type 1 (NF1), and the Carney-Stratakis dyad [2,4,5,11]. The patients presented herein were diagnosed with PGL 4 syndrome caused by SDHB mutation, which possesses unique characteristics and prognostic factors impacting perioperative management. These include the earlier onset of the disease, higher mortality rates, secretion of dopamine and norepinephrine rather than epinephrine, a higher rate of extra-paraganglia neoplasms, and a tendency to metastasize [2,12,13]. Understanding the natural history and prognosis related to a specific mutation can guide and enhance the patients’ postoperative management. Notably, IHC in one of the patients was negative for SDHB staining when subsequent genetic testing revealed a pathologic SDHB mutation, highlighting the importance of genetic testing in all cases of extra-adrenal paragangliomas.

Surgeons should suspect a hereditary syndrome associated with paraganglioma when presented by any of several clinical hallmarks. Classically, familial paragangliomas encompass a younger age at diagnosis, the occurrence of extra-adrenal and multifocal disease, and the presence of associated non-paraganglial tumors. However, the phenotype can vary significantly depending on the genotype [2]. Earlier clinical onset of the disease is strongly associated with SDHB and VHL mutations. Indeed, up to 67% of SDHB cases manifest by the age of 30 compared to 20% in sporadic cases [11,13]. The presented patients were diagnosed at 17- and 37-years-old, which raised a strong suspicion of an identifiable deleterious genetic mutation. Multifocal manifestation is another typical hallmark of a hereditary syndrome reported in up to 80% of cases, although this rate varies by genotype [2,11]. In both of our patients, the paraganglioma was unifocal and located in the left paraaortic region. Presentation with multiple lesions is more typical of parasympathetic non-functional paragangliomas located in the neck and skull base, likely due to a typically more indolent course [14]. In the study of Neuman et al., none of the 12 paraganglioma patients with SDHB mutation had multifocal disease compared to 35-40% of SDHD and VHL patients [11]. It is unclear if the presence of a single paraganglioma in the presented cases can be explained by the specific association of SDHB mutation with solitary tumors or can be attributed to the tendency of SDHB-mutated paragangliomas to secrete catecholamines and prompt early diagnosis [5,12]. As only one of the cases presented symptomatically, it is likely that both possibilities were at play in our patients having a single tumor. Interestingly, patient 1, with a more aggressive variant and GAPP score of 8, presented earlier and with prominent symptoms of catecholamine secretion whereas patient 2, diagnosed with a low GAPP score, paraganglioma presented later in life as an asymptomatic, incidental finding [9]. Close follow-up with imaging for both patients will be imperative not only for detecting a recurrence but also for identifying new primary lesions.

Other unique PGL 4 clinical features include rare association with non-paraganglioma tumors, such as GIST, RCC, and pituitary tumors, and the highest potential for metastatic disease among all hereditary PGL syndromes [2,12]. In one study, 34% of SDHB mutations had metastatic disease compared to 0% among patients with SDHD and 3% of sporadic cases [12]. Manneli et al. reported similar findings - a 20.8% metastatic disease rate in SDHB patients compared to 4.3% in SDHD; 4.2% in VHL; and no malignant or metastatic cases in RET, NF1, and SDHC [15]. Patient 1 had a poorly differentiated paraganglioma with aggressive features including vascular invasion, positive resection margin, and high GAPP score of 8, but no LN or distant metastases. Therefore, we scheduled frequent follow-ups including physical exams, imaging, plasma metanephrines levels, and ctDNA every six months, with no signs of recurrence to date. While the use of ctDNA for malignant paraganglioma is under investigation, it has shown an impressive correlation with relapse prediction and diagnosis in other types of tumors, including colon, breast, and lung cancers [16]. In this case, negative ctDNA levels at one- and six-months postoperatively have supported less frequent imaging allowing a decrease in radiation exposure to this young patient. We did not obtain ctDNA testing in the second patient as his tumor demonstrated favorable pathologic features.

Interestingly, despite an autosomal dominant pattern of inheritance in HPPS, neither patient had a known family history of paraganglioma or associated tumors. Moreover, thus far, the three family members of patient 1 who were found to have the same mutation have no obvious phenotypical features and are significantly older than the index patient. The concept of genetic predisposition may be confusing for patients and their families, particularly with genetic disorders with low penetrance. Although the SDHB inheritance is autosomal dominant (the offspring of the carrier has a 50% chance of getting the mutation), the lifetime penetrance of SDHB mutation is about 22% and age-related, translating into only a one in five chance for carriers to exhibit the disease phenotype [2,17,18]. Thus, cascade genetic testing is indicated in all paraganglioma cases associated with germline SDHx mutations.

Surgical resection remains the main management modality for both benign and malignant paragangliomas and allows for control of hormonal secretion, symptom resolution, and improvement in survival [2]. Surgery for abdominal paragangliomas can be performed via retroperitoneal or transabdominal approaches using open or minimally invasive techniques. No high-quality data from randomized controlled trials exist to support either approach due to disease rarity. Some authors oppose minimally invasive surgery (MIS) in dopamine-secreting tumors with SDHB mutation due to the high risk of malignancy [19,20]. Nevertheless, the robotic approach can offer significant advantages over open surgery in the retroperitoneum including better visualization and precision of dissection and the ability to access anatomically challenging areas. In case 1, primary tumor resection with LN dissection was performed completely robotically, with adequate oncologic outcomes while keeping MIS advantages such as fast recovery and low postoperative morbidity. While a positive resection margin was identified postoperatively, this was expected due to tumor adjacency to the aorta and left renal vessels. R0/R1 resection is acceptable and considered complete for many retroperitoneal tumors due to the frequent large size of these tumors and the anatomic complexity of the retroperitoneum. The operative course of case 2 illustrates the importance of a high degree of surgical expertise in both the open and robotic approach to allow the seamless transition from one technique to the other in cases of unexpected surgical challenges.

## Conclusions

The presented cases demonstrate the feasibility of MIS for treating selected patients with non-metastatic paraganglioma associated with HPPS. They underscore the importance of postoperative genetic testing for all paraganglioma cases to customize future management and screening approaches for these uncommon patients. A comprehensive understanding of the natural history and varied presentations within a single gene mutation for these tumors is vital for predicting outcomes and creating tailored treatment and monitoring strategies for patients with paragangliomas.
